# Case of Successful Amputation at the Shoulder-Joint, Performed under Very Unpromising Circumstances

**Published:** 1818-01

**Authors:** Joseph M'Carogher

**Affiliations:** Surgeon R. N.


*Case of successful Amputation at the Shoulder-joint, performed under very unpromising Circumstances.* Communicated by JOSEPH M'CAROGHER, Esq. Surgeon R. N.

THE subject of this case was a seaman, of a spare habit, and about fifty years of age. On the 3d day of November, ——, whilst in the act of reloading one of the main-deck guns, by its accidental explosion, he had his right fore-arm blown off, and the humerus contused and shattered close up to the insertion of the deltoid muscle. Two splinters of wood entered nigh to the head of the bone, at the inner margin of the deltoid, and penetrated across for three inches into the pectoral muscle.

The face was much scorched and loaded with powder. There was also a wound of an inch long beneath the right eye (which latter suffered much, from receiving a great portion of the fire), and another wound of three inches, over the right parietal bone.

Though this man, when put on the table, was almost in a lifeless state, I decided on amputating immediately; and thus giving him every chance in my power.—After removing the splinters, and making compression on the artery, from *above* the clavicle, with a piece of hard dry sponge, I operated in the following manner:

With a strong scalpel I made a semilunar incision along the anterior and posterior margin of the deltoid, so as to raise the whole of it, from its insertion up to the joint. Having gone so far, and secured two of the circumflex arteries which sprang, I divided the anterior part of the capsular ligament, and the tendon of the long head of the biceps, to such extent as the raising of the deltoid would admit;—having turned the arm out, so as to make a partial dislocation of the bone forward, with one stroke of the amputating knife I cut through the integuments and muscles beneath, secured the artery, and then dissected out the bone;—having washed and sponged the wound well, I found it requisite to secure two small branches more, making in all five. The flap of the deltoid was brought down, secured by three stitches, and dressed in the usual manner. He was then put to bed, and took an anodyne.

For several days (although he lost but very little blood during the operation), the arterial action was so languid, and the heat of the skin so much below the natural standard, that I judged it necessary to administer wine in small quantities, every two or three hours. This, at length, brought on the accustomed healthy action. Symptomatic fever was kept under by purgatives and the constant liberal use of barley water, acidulated with crem. tartar. To the face was applied lotio litharg. acet.—The succeeding inflammation of the right eye was very violent; so much so, that, at one time, a complete opacity of the lucid cornea had taken place: but, by the use of repeated blisters to the temple, scarification of the vessels of the adnata, and cold saturnine lotions, it was subdued; leaving only a small speck, which diminished daily. The patient was quite well, and all his wounds cured in two months.

